# Early transcriptional states of spermatogonia and marker expressions in the prepubertal human testis following chemotherapy-induced depletion

**DOI:** 10.1093/humrep/deaf103

**Published:** 2025-06-07

**Authors:** Hajar Ba Omar, Justine Stevens, Anu Haavisto, Yanhua Cui, Femke Harteveld, Yifan Yang, Ragnar Bjarnason, Patrik Romerius, Mikael Sundin, Ulrika Norén Nyström, Cecilia Langenskiöld, Hartmut Vogt, Per Frisk, Kaisa Vepsäläinen, Cecilia Petersen, Lina Cui, Jingtao Guo, Kirsi Jahnukainen, Jan-Bernd Stukenborg

**Affiliations:** NORDFERTIL Research Lab Stockholm, Childhood Cancer Research Unit, Department of Women’s and Children’s Health, Karolinska Institutet, and Karolinska University Hospital, Solna, Sweden; NORDFERTIL Research Lab Stockholm, Childhood Cancer Research Unit, Department of Women’s and Children’s Health, Karolinska Institutet, and Karolinska University Hospital, Solna, Sweden; Department of Gynecology and Obstetrics, University Medical Centre of the Johannes Gutenberg University, Mainz, Germany; NORDFERTIL Research Lab Stockholm, Childhood Cancer Research Unit, Department of Women’s and Children’s Health, Karolinska Institutet, and Karolinska University Hospital, Solna, Sweden; Department of Psychology, University of Helsinki, Helsinki, Finland; Faculty of Education and Welfare Studies, Åbo Akademi University, Vasa, Finland; NORDFERTIL Research Lab Stockholm, Childhood Cancer Research Unit, Department of Women’s and Children’s Health, Karolinska Institutet, and Karolinska University Hospital, Solna, Sweden; NORDFERTIL Research Lab Uppsala, Department of Organismal Biology, Uppsala University, Uppsala, Sweden; NORDFERTIL Research Lab Stockholm, Childhood Cancer Research Unit, Department of Women’s and Children’s Health, Karolinska Institutet, and Karolinska University Hospital, Solna, Sweden; NORDFERTIL Research Lab Stockholm, Childhood Cancer Research Unit, Department of Women’s and Children’s Health, Karolinska Institutet, and Karolinska University Hospital, Solna, Sweden; Children’s Medical Centre, Landspítali University Hospital, Reykjavik, Iceland; Department of Paediatrics Faculty of Medicine, University of Iceland, Reykjavik, Iceland; Department of Paediatric Oncology and Haematology, Clinical Sciences, Lund University, Barn- och ungdomssjukhuset Lund, Skånes universitetssjukhus, Lund, Sweden; Division of Paediatrics, Department of Clinical Science, Intervention and Technology, Karolinska Institutet, Stockholm, Sweden; Section of Haematology, Immunology and HCT, Astrid Lindgren Children’s Hospital, Karolinska University Hospital, Stockholm, Sweden; Division of Paediatrics, Department of Clinical Science, Umeå University, Umeå, Sweden; Department of Paediatric Oncology, The Queen Silvia Children’s Hospital, Gothenburg, Sweden; Division of Children’s and Women’s Health, Department of Biomedical and Clinical Sciences, Crown Princess Victoria's Children’s Hospital, Linköping University, Linköping, Sweden; Pediatric Haematology & Oncology Children’s University Hospital, Uppsala, Sweden; Department of Paediatrics, Kuopio University Hospital, Kuopio, Finland; NORDFERTIL Research Lab Stockholm, Childhood Cancer Research Unit, Department of Women’s and Children’s Health, Karolinska Institutet, and Karolinska University Hospital, Solna, Sweden; State Key Laboratory of Stem Cell and Reproductive Biology, Institute of Zoology, Chinese Academy of Sciences, Beijing, China; Beijing Institute for Stem Cell and Regenerative Medicine, Beijing, China; University of Chinese Academy of Sciences, Beijing, China; State Key Laboratory of Stem Cell and Reproductive Biology, Institute of Zoology, Chinese Academy of Sciences, Beijing, China; Beijing Institute for Stem Cell and Regenerative Medicine, Beijing, China; University of Chinese Academy of Sciences, Beijing, China; NORDFERTIL Research Lab Stockholm, Childhood Cancer Research Unit, Department of Women’s and Children’s Health, Karolinska Institutet, and Karolinska University Hospital, Solna, Sweden; New Children's Hospital, Paediatric Research Centre, University of Helsinki and Helsinki University Hospital, Helsinki, Finland; NORDFERTIL Research Lab Stockholm, Childhood Cancer Research Unit, Department of Women’s and Children’s Health, Karolinska Institutet, and Karolinska University Hospital, Solna, Sweden; NORDFERTIL Research Lab Uppsala, Department of Organismal Biology, Uppsala University, Uppsala, Sweden

**Keywords:** testis, prepubertal, single-cell gene expression analysis, immunohistochemistry, spermatogonia

## Abstract

**STUDY QUESTION:**

Which spermatogonial differentiation states are present in prepubertal testes under normal conditions and following chemotherapy-induced depletion of spermatogonia in paediatric patients with cancer?

**SUMMARY ANSWER:**

Single-cell transcriptomic analysis reveals that only undifferentiated spermatogonia are present in prepubertal boys, while differentiated states emerge during puberty, with reduced protein expression of advanced spermatogonial markers observed in younger patients, those treated with alkylating agents, or those with a diminished spermatogonial pool.

**WHAT IS KNOWN ALREADY:**

Paediatric oncology treatments often involve gonadotoxic therapies that can impair spermatogonial stem cells, increasing the risk of subfertility. While five distinct spermatogonial subpopulations have been identified in adult testes via single-cell RNA sequencing, their presence in prepubertal testes of childhood cancer patients remains to be confirmed through marker protein expression.

**STUDY DESIGN, SIZE, DURATION:**

Gene expression profiles of spermatogonial subpopulations were investigated using single-cell RNA sequencing data from six testicular samples of healthy boys aged 0–17 years. Protein expression patterns were examined via immunofluorescence staining in 14 biobank control samples (median age: 4.9 years; range: 0.6–13.1 years) and in 31 prepubertal testicular tissue samples of paediatric patients with cancer (median age: 6.8 years; range: 0.7–13.1 years).

**PARTICIPANTS/MATERIALS, SETTING, METHODS:**

Gene expression profiles of UTF1 (states 0–1), ID4 (states 0–1), PIWIL4 (states 0–1), FGFR3 (states 0–2), and KIT (state 4), were analysed in testicular cells of paediatric origin obtained from our previously published open-access data source (GSE134144 and GSE120508). The protein expression of these spermatogonial subpopulation markers was evaluated by counting immunofluorescence-positive cells per analysed area. Marker expression was correlated with prior chemotherapy exposure and spermatogonia numbers. Exposure to alkylating agents was quantified as the cumulative cyclophosphamide equivalent dose (CED), and anthracycline exposure as the cumulative doxorubicin isoequivalent dose equivalents (DIE). A depleted spermatogonia pool was defined as having S/T *Z*-scores lower than −7 SD.

**MAIN RESULTS AND THE ROLE OF CHANCE:**

Transcriptomic analysis confirmed that germ cells in the prepubertal testis consist solely of undifferentiated spermatogonia. The expression of KIT protein, defining differentiated spermatogonia, was positively correlated with age (*P* < 0.001). A reduction in the number of spermatogonia expressing ID4 protein was associated with higher CED (*P* = 0.001), and spermatogonia expressing KIT protein with higher CED and DIE exposure (*P* = 0.005, and *P* = 0.035, respectively). A depleted spermatogonia pool (S/T *Z*-score <−7 SD) correlated with fewer spermatogonia expressing ID4 (*P* = 0.033), FGFR3 (*P* = 0.050), and KIT (*P* = 0.051) proteins. These results indicate that distinct protein expression patterns were observed following chemotherapy-induced reduction of the spermatogonial pool, with reduced expression of ID4, FGFR3, and KIT proteins. Numbers of spermatogonia positive for markers indicating more naïve, undifferentiated states, such as UTF1 and PIWIL4, did not correlate with spermatogonial pool reduction.

**LIMITATIONS, REASONS FOR CAUTION:**

The study population was heterogeneous in terms of age and treatment exposure. Moreover, the impact of specific cancer treatments could not be individually assessed. Limited tissue availability reduced the statistical power of the study, and repeated double or triple immunofluorescence staining could not be performed. As a result, the correlations between the expression of different spermatogonial markers can only be considered indicative trends. Child testicular control tissue samples were considered normal for inclusion if no testicular pathology was reported. However, detailed information on prior medical treatments or testicular volumes for the patients in this biobank was unavailable.

**WIDER IMPLICATIONS OF THE FINDINGS:**

Our observations suggest that alkylating agents have dose-dependent effects on all spermatogonial subpopulations. However, spermatogonial subtypes expressing the protein markers UTF1 and PIWIL4 were more resistant to chemotherapy-induced depletion of the spermatogonial pool, potentially representing true reserve stem cells. The identification of reserve stem cells could provide a valuable method for evaluating the fertility potential of testicular tissue collected for fertility preservation in prepubertal and peripubertal boys.

**STUDY FUNDING/COMPETING INTEREST(S):**

This study was supported by grants from the Swedish Childhood Cancer Fund (PR2019-0123; PR2022-0115; TJ2020-0023) (J.-B.S.), Finnish Cancer Society (K.J.), Finnish Foundation for Paediatric Research (K.J.), Swedish Research Council (2018-03094; 2021-02107) (J.-B.S.), and Birgitta and Carl-Axel Rydbeck’s Research Grant for Paediatric Research (2020-00348; 2020-00335; 2021-00073; 2022-00317, 2024-00255) (J.-B.S., K.J.). Y.C. and Y.Y. received a scholarship from the Chinese Scholarship Council. J.S. was supported by a grant from Mary Béves Foundation for Childhood Cancer Research. H.B.O. was supported by the Sultan Qaboos University in Oman. The authors declare no competing interests.

**TRIAL REGISTRATION NUMBER:**

N/A.

## Introduction

The production of sperm after puberty relies on the existence and functionality, as well as integrity, of spermatogonia. Reference values for spermatogonial numbers during testicular maturation have been established, indicating a decline from birth to 3 years of age, followed by a gradual increase until age 7 years, after which spermatogonial numbers remain stable (Masliukaite *et al.*, 2016). These physiological processes can be disrupted by genetic or endocrine disorders, as well as medical interventions such as chemo- or radiation therapy, resulting in partial to complete depletion of spermatogonia (Stukenborg *et al.*, 2018; Funke *et al.*, 2021; Kanbar *et al.*, 2021; Benninghoven-Frey *et al.*, 2022; Masliukaite *et al.*, 2023; Lahtinen *et al.*, 2024). To accurately evaluate the adverse effects, developmental variations in spermatogonia numbers need to be controlled. For this purpose, reference means for spermatogonia numbers across human development have been established, enabling the calculation of *Z*-scores (Funke *et al.*, 2021).

The spermatogonial stem cell (SSC) population has traditionally been classified into *A*_dark_ and *A*_pale_ subtypes based on morphological characteristics (Clermont, 1963). *A*_dark_ SSCs have been proposed to represent a reserve stem cell population that may replenish the spermatogonial pool following insult, while *A*_pale_ SSCs are proposed to represent a progenitor population committed to continuously proliferate and differentiate. Recent studies have focused on refining the human spermatogonia into subpopulations based on functional aspects (Di Persio and Neuhaus, 2023), or using protein expression profiles for spermatogonia, such as PLZF (ZBTB16), undifferentiated embryonic cell transcription factor 1 (UTF1), survival time-associated PHD finger in ovarian cancer 1 (SPOC1; PHF13), GFRA1, fibroblast growth factor receptor 3 (FGFR3), c-Kit receptor tyrosine kinase (KIT), and doublesex- and mab-3-related transcription factor 1 (DMRT1) (von Kopylow *et al.*, 2012). For instance, advances in scRNA-seq studies have identified five distinct spermatogonial subpopulations, characterized by their expression profiles. These range from the most naïve, reserved undifferentiated state (state 0), through intermediate states undergoing sequential changes (states 1, 2, and 3), to the most differentiated state committed to entering meiosis (state 4) (Guo *et al.*, 2018, 2020, 2021). A direct comparison with the morphological classification using *A*_dark_, *A*_pale_, and B spermatogonia is challenging. However, *A*_dark_ spermatogonia, historically regarded as reserve stem cells with low proliferative activity, most closely correspond to state 0. In contrast, *A*_pale_ spermatogonia best align with states 1–3, encompassing both proliferative and differentiative phases of spermatogonial development. Type B spermatogonia, which are mitotically active and committed to differentiation, correspond to state 4 cells. Observations suggest that during prepubertal stages, the testes maintain a pool of undifferentiated, largely quiescent state 0 germline stem cells, which begin to mature during puberty and give rise to the five spermatogonial states observed in adult testes. Remarkably, scRNA-seq and protein validation have not provided evidence for a simple molecular basis underlying the histologically distinct populations of *A*_dark_ and *A*_pale_ spermatogonia. These findings underscore the need for further proteomic and functional analyses, as well as spatial data, to enhance our understanding of the mechanisms governing early spermatogonial maturation. The maintenance and maturation of early spermatogonia are particularly critical for understanding the effects of gonadotoxic therapies, as the potential for sperm production recovery following cytotoxic damage depends on the ability of mitotically quiescent spermatogonia to survive and generate differentiating spermatogonia. Similarly, current strategies to preserve the reproductive potential of prepubertal patients rely on the quantity and functionality of SSC reserves within tissue samples.

This study integrated previously published open-access scRNA-seq data of spermatogonia from pre- and peripubertal healthy testes with immunofluorescence staining of early spermatogonial markers. Testicular tissue samples were obtained from an internal biobank of specimens without known pathology and from childhood patients with cancer. The aim was to characterize spermatogonial differentiation states in pre- and peripubertal testicular tissue and identify which subpopulations persist following chemotherapy-induced reduction in childhood patients with cancer, potentially representing a quiescent reserve of the SSC pool.

## Materials and methods

### Ethical approval

Ethical approval for the use of testicular tissues was obtained from the Regional Ethics Board in Stockholm (archived sample collection of the Department of Pathology at Karolinska University Hospital: Dnr 2014-267-31/4, and Dnr 2024-01273-02; NORDFERTIL sample collection: Dnr 2013-2129-31-3, and Dnr 2021-04277), National Ethics Board of Iceland, Reykjavik (VSN 15-002), and Ethics Board of the University of Helsinki (426/13/03/03/2015).

### Sample collection

**Figure 1.**
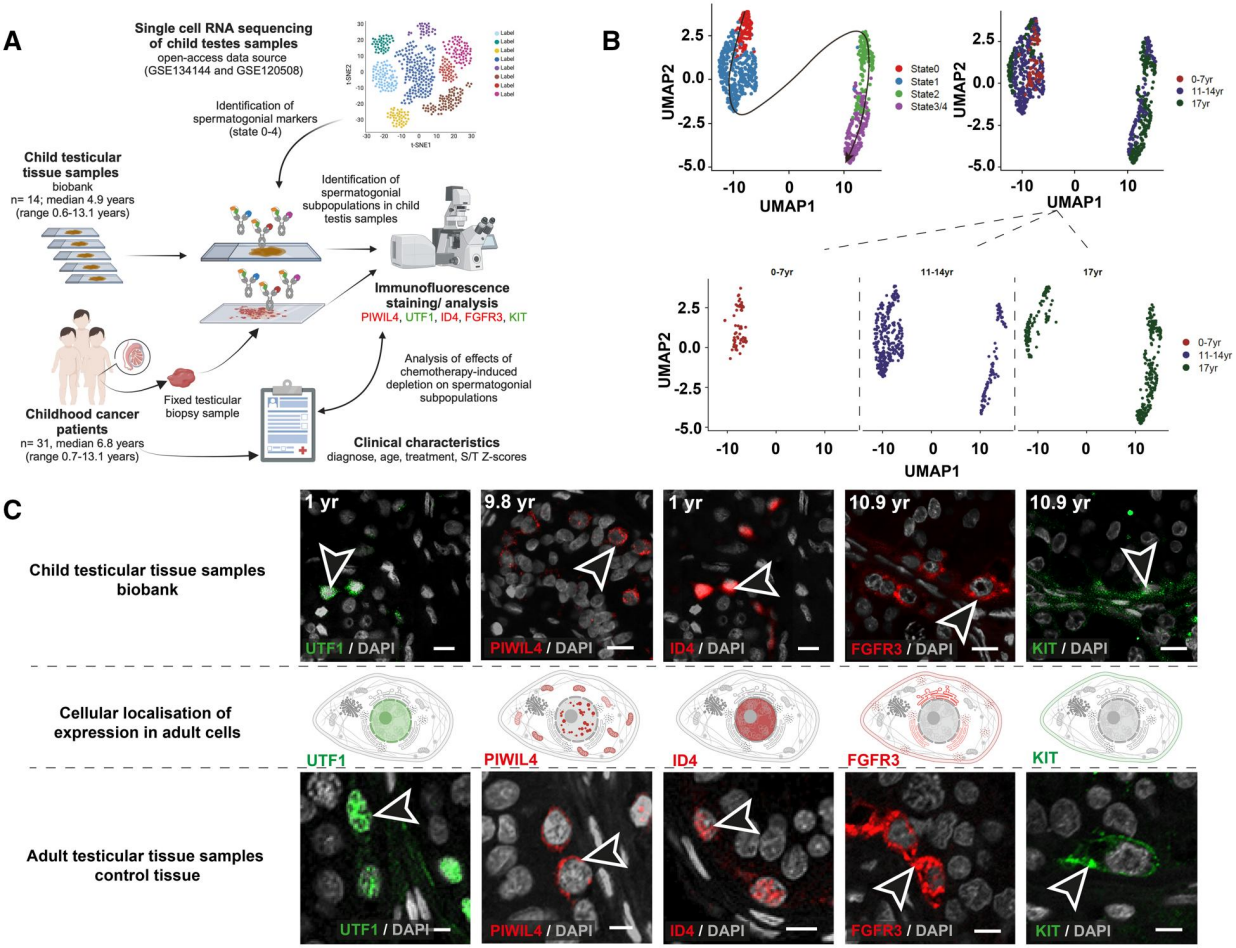
**Human spermatogonial marker expression across developmental stages.** (**A**) Schematic illustration of the experimental workflow (Created in BioRender^®^. Stukenborg, J. (2025) https://BioRender.com/1zgv318). (**B**) UMAP-based dimensional reduction representation of combined single-cell transcriptome data from human child testicular cells (n = 786). Each dot represents a single cell, coloured by either age (0–7 years, 11–14 years, or 17 years) or the corresponding spermatogonial differentiation state (0–4). (**C**) Schematic illustration of the expression localization of UTF1 (green; nucleus), PIWIL4 (red; mitochondria, nucleoplasm), ID4 (red, nucleoplasm), FGFR3 (red; endoplasmic reticulum, secreted into the cytoplasm, plasma membrane (surface protein/receptor)), and KIT (green; plasma membrane) in adult cells, as described by the Human Protein Atlas. Representative immunofluorescence staining images show spermatogonia expressing UTF1 (green), PIWIL4 (red), ID4 (red), FGFR3 (red), and KIT (green) in both child and adult testicular samples (indicated by arrows). Cell nuclei are counterstained with DAPI (grey). Scale bars: 10 µm. UMAP, uniform manifold approximation and projection; yr, years; UTF1, undifferentiated embryonic cell transcription factor 1; PIWIL4, PIWI-like protein 4; ID4, inhibitor of DNA binding 4; FGFR3, fibroblast growth factor receptor 3; KIT, tyrosine kinase receptor.

Testicular biopsy samples were obtained from pre- and peripubertal patients (n = 31; age range 0.6–13.1 years; identified as P1–31, Supplementary Table S1, Fig. 1A). The samples were obtained from patients who participated in the NORDFERTIL fertility preservation project (NORDFERTIL sample collection). These patients were considered at high risk of treatment-associated infertility (allogeneic/autologous haematopoietic cell transplantation or radiotherapy involving the testis), in accordance with the guidelines on fertility preservation of the Nordic Society of Paediatric Haematology and Oncology (https://pho.barnlakarforeningen.se/wp-content/uploads/sites/20/2019/11/PM-SALUB-Fertility-preservation-for-boys-and-young-men-1.2.pdf). Exposure to cyclophosphamide equivalent dose (CED) or doxorubicin isoequivalent dose equivalents (DIE) was not an exclusion criterion. Exclusion criteria for testicular biopsy were pre-existing spermatogenesis (testicular volumes >10 ml by orchidometer) and a high bleeding or infection risk. The clinical characteristics of the 31 patients, including age, diagnosis, and cancer therapy prior to testicular biopsy, were reported by their designated physicians (Supplementary Table S1). Alkylating agent exposure was calculated as the cumulative CED (Green *et al.*, 2014) and anthracycline exposure as the cumulative DIE using a conversion factor of 1.0 for doxorubicin, 0.67 for epirubicin, 5.0 for idarubicin, and 4.0 for mitoxantrone (Shankar *et al.*, 2008) as well as 0.6 for daunorubicin (Feijen *et al.*, 2019) (Supplementary Table S1). Verbal and written consent for participation in the research project were obtained from the patients and/or parents for all samples. To preserve fertility, 20% of the testicular volume of one testis was obtained. Two-thirds of these 20% were cryopreserved for future clinical fertility preservation; the remaining one-third were transported as fresh tissue to the NORDFERTIL research laboratory at Karolinska Institutet for research and were included in this study. Approximately 5% of this fresh testicular tissue was immediately fixed in 4% paraformaldehyde (PFA, HL96753.1000 Histolab, Askim, Sweden) and embedded in paraffin for histological analysis. Sections of paraffin-embedded testicular tissue samples (n = 14, age range 0.6–13.1 years; identified as P32–45, Supplementary Table S1, Fig. 1A) obtained from the biobank at the Department of Pathology at Karolinska University Hospital, without underlying pathologies served as controls.

### Immunofluorescence staining

For immunofluorescence analysis, three sections per sample were subjected to heat-mediated antigen retrieval at 95 °C in 1x Tris–EDTA (T1503, Sigma–Aldrich, St Louis, MO, USA) at a pH of 9 for 24 min. Subsequently, the samples were blocked in Tris-buffered saline (TBS) supplemented with 10% normal donkey serum (017-000-121, Jackson Immuno Research, West Grove, PA, USA) and 1% bovine serum albumin (A2153, Sigma–Aldrich). The primary antibodies of DEAD-box helicase 4 (DDX4), UTF1, PIWI-like protein 4 (PIWIL4), Inhibitor of DNA Binding 4 (ID4), FGFR3, and KIT (Supplementary Table S2; Supplementary Fig. S1, Fig. 1C) were diluted in blocking buffer, and the slides were incubated overnight at 4 °C. Mouse, goat, or rabbit IgGs were used as negative controls according to the host of the primary antibody used (Supplementary Table S2). After three washing steps in TBS for 5 min each, the samples were incubated at room temperature with fluorescent-conjugated secondary antibodies (Supplementary Table S2, Supplementary Fig. S1), and 4,6-diamidino-2-phenylindole (DAPI) (135-1303, Bio-Rad, Hercules, CA, USA), which were diluted in blocking buffer. The slides were mounted with an anti-fade mounting medium (P36931; Invitrogen, Waltham, MA, USA). Immunofluorescence images were obtained using a confocal microscope (LSM700, Zeiss, Jena, Germany).

### Evaluation of immunofluorescence staining

UTF1, PIWIL4, ID4, FGFR3, and KIT expressing cells (Supplementary Fig. S2, Fig. 1C) were counted using the ImageJ Particle Analyzer. Positive signals for each marker were identified in cells with nuclei counterstained with DAPI, followed by measurement of the tubular area. All cord and tubule areas were then manually annotated and recorded in square millimetres (mm^2^).

### Spermatogonial numbers per round tubules (S/T) and Z-score calculations

For the evaluation of spermatogonial numbers, at least two independent non-consecutive sections of testicular tissue obtained on the day of biopsy, were immunostained against the germ cell marker DDX4, as described above. Spermatogonia were identified based on their morphology (size, shape), location (Paniagua and Nistal, 1984), and DDX4 expression. Employing a blinded approach, all-round tubular cross-sections within the tissue sections were quantified. Mean spermatogonial numbers per round tubular cross-section (S/T) were assessed. To control physiological variation in spermatogonial numbers during development, age-independent *Z*-scores were calculated for S/T (Supplementary Table S1) using the reference means (Funke *et al.*, 2021). Part of the S/T *Z*-score data included in this study has been published in two recent publications (Funke *et al.* 2021; Cui *et al.*, 2024).

### Single-cell gene expression analysis of spermatogonial markers

Human child testis samples were prepared for scRNA-seq as previously described (Guo *et al.*, 2020). In brief, scRNA-seq was performed, sequencing library prepared, libraries sequenced, and scRNA-seq data processed as previously reported (Guo *et al.*, 2020). The raw gene expression matrices from human child testis samples were obtained from the Gene Expression Omnibus (GEO) (Clough and Barrett, 2016) under accession numbers GSE134144 and GSE120508 and were reprocessed following previously established protocols. For downstream analysis, all available published data were combined for quality control. Features with fewer than 500 or more than 8000 counts, unique molecular identifiers with fewer than 800 or more than 20 000 counts, and cells with more than 25% mitochondrial mapping were filtered out. Following this, principal component analysis (PCA) and uniform manifold approximation and projection analysis were performed using 2000 highly variable genes. Each cluster was annotated based on the top 20 genes identified by the FindAllMarkers (Hao *et al.*, 2024) function with default parameters. Visualization of the results was achieved using the VlnPlot and FeaturePlot functions in Seurat (Hao *et al.*, 2024).

### Statistical analysis

All data are presented as median and range. Partial Spearman’s rank correlation analyses were performed; first between the different spermatogonia marker expressions and age; second between the different spermatogonia marker expressions, CED, DIE, and S/T *Z*-scores, when controlled for age. Patients were divided into two groups based on their S/T *Z*-scores: patients with severely depleted spermatogonia (<−7 SD, n = 10) and patients without a depleted spermatogonial pool (>−7 SD, n = 35). Mann–Whitney *U*-tests were applied to determine if there were statistically significant differences between the patient groups with and without depleted spermatogonia pool. IBM SPSS Statistics 29.02.0 (Armonk, NY, USA) or GraphPad Prism software version 10.2.3 for Windows (GraphPad Software, San Diego, CA, USA) were employed to conduct the statistical analyses. All significance tests were two-tailed and a *P*-value of <0.05 was considered statistically significant.

## Results

### Identification of known spermatogonial states in child testis

**Figure 2.**
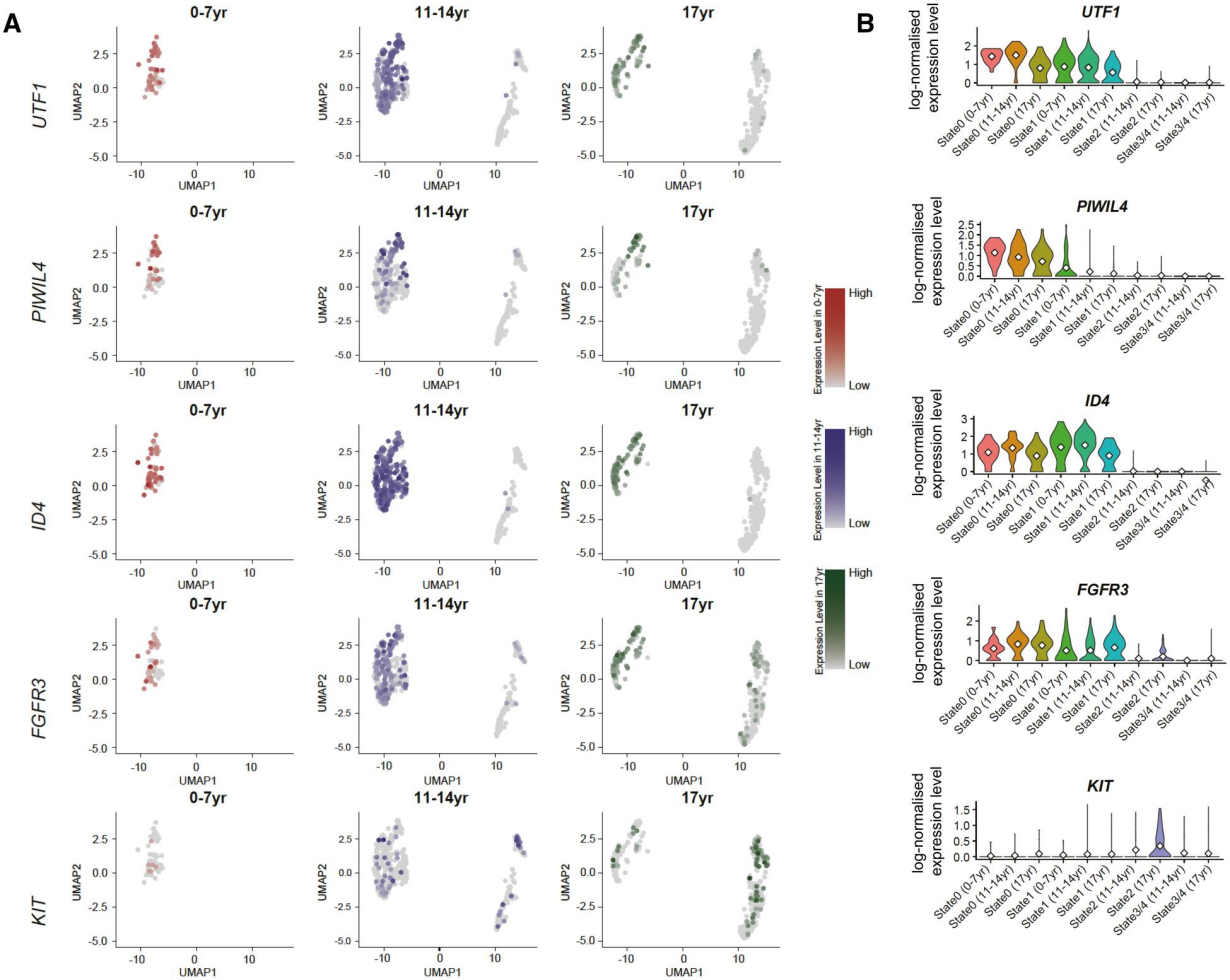
**Single-cell transcriptome profiling of healthy child testis.** (**A**) UMAP-based dimensional reduction representation of combined single-cell transcriptome data from human child testicular cells (n = 786). Each dot represents a single cell, coloured by age (0–7 years [red], 11–14 years [blue], or 17 years [green]) or the corresponding spermatogonial marker expression (UTF1, PIWIL4, ID4, FGFR3, and KIT). (**B**) Spermatogonial marker expression (UTF1, PIWIL4, ID4, FGFR3, and KIT) is shown as normalized expression level based on differentiation state (0–4) and age. UMAP, uniform manifold approximation and projection; yr, years; UTF1, undifferentiated embryonic cell transcription factor 1; PIWIL4, PIWI-like protein 4; ID4, inhibitor of DNA binding 4; FGFR3, fibroblast growth factor receptor 3; KIT, tyrosine kinase receptor.

To characterize the spermatogonial states in child testis, we used clusters previously characterized in adult testis. This analysis yielded five distinct clusters with high similarity to the clusters/states previously described in adult testis (Guo *et al.*, 2018) (Fig. 1B). None of the clusters/states (including state 0) consisted of cells derived from a particular donor. Pseudotime analysis revealed a wave-like progression from state 0 to state 4. Samples from boys aged 0–7 years consisted exclusively of cells in states 0 and 1, while samples from individuals aged 11–14 years and 17 years included cells in all states 0–4, indicating the onset of spermatogenesis (Fig. 2). Clustering analyses defined gene expression signatures associated with each state that were consistent with those previously reported in the adult testis (Guo *et al.*, 2018). *PIWIL4* expression was predominantly restricted to state 0–1 spermatogonia and exhibited a stable and consistent pattern across all paediatric age groups. *UTF1*, which is expressed in state 0–1 similarly to *PIWIL4*, demonstrated a significant expansion within the 11- to 14-year age group compared with both younger and older cohorts. Likewise, *ID4* expression, while expressed in state 0–1 similarly to *PIWIL4* and *UTF1*, showed specific enrichment within the expanded middle and lower segments of state 1 in the 11- to 14-year age group. *FGFR3* expression displayed significant variability between the 17-year-old sample and younger cohorts. High *FGFR3* expression was exclusively observed in the 17-year-old sample, localized to states 2–3. Similarly, *KIT* expression was predominantly enriched in states 2 and 3/4 and restricted to the 11- to 14- and 17-year age groups, aligning with their established roles in more differentiated spermatogonial populations.

### In situ observation of early spermatogonial states via protein immunofluorescence in child testis

The protein expression of early spermatogonial markers (UTF1, PIWIL4, ID4, FGFR3, and KIT), displaying differential expression across early states (0–4), was analysed using immunofluorescence staining (Fig. 1C, Supplementary Fig. S2). In samples with a non-depleted spermatogonia pool (S/T *Z*-scores ≥−7 SD), spermatogonia expressing all five markers were predominantly localized at the periphery of the seminiferous cords. However, in testicular samples from patients with a depleted spermatogonial pool (S/T *Z*-scores <−7 SD), the expression of spermatogonial markers was less distinct (Supplementary Fig. S2). Protein expression of KIT showed a positive correlation with patient age (Table 1) and was frequently detected after the age of 10 years but rarely earlier. The other early spermatogonia markers showed no age dependency (Fig. 1, Supplementary Fig. S3, Table 1).

**Table 1.** Correlations between spermatogonia marker expression, clinical characteristics, and spermatogonia quantity in 45 prepubertal testicular samples, controlled for age.

Variable	UTF1		PIWIL4		ID4		FGFR3		KIT	
	ρ	*P*-value	ρ	*P*-value	ρ	*P*-value	ρ	*P*-value	ρ	*P*-value
**Clinical characteristic**										
Age^a^	0.059	0.719	0.101	0.509	0.177	0.249	0.007	0.963	0.519	**<0.001**
CED	−0.034	0.835	−0.196	0.203	−0.483	**0.001**	−0.261	0.095	−0.420	**0.005**
DIE	0.224	0.170	−0.132	0.395	−0.267	0.083	0.012	0.940	−0.318	**0.035**
**Spermatogonia quantity**										
S/T *Z*-score	−0.125	0.448	0.102	0.509	0.453	**0.002**	0.200	0.205	0.465	**0.001**
Below reference^b^	0.198	0.227	−0.029	0.853	−0.352	**0.021**	−0.035	0.827	−0.428	**0.004**
Depleted^c^	−0.093	0.35	−0.222	0.148	−0.326	**0.033**	−0.304	**0.050**	−0.364	**0.051**
**Spermatogonia marker expression**										
UTF1	–	–	0.285	0.079	0.111	0.500	0.495	**0.002**	−0.356	**0.026**
PIWIL4	0.285	0.079	–	–	0.546	**<0.001**	0.674	**<0.001**	−0.008	0.960
ID4	0.111	0.500	0.546	**<0.001**	–	–	0.658	**<0.001**	0.209	0.178
FGFR3	0.495	**0.002**	0.674	**<0.001**	0.658	**<0.001**	–	–	0.097	0.539
C-KIT	−0.356	**0.026**	−0.008	0.960	0.209	0.178	0.097	0.539	–	–

ρ, Spearman correlation coefficient; CED, cumulative cyclophosphamide equivalent dose; DIE, doxorubicin isoequivalent dose; S/T, spermatogonia numbers per round tubular cross-section; UTF1, undifferentiated embryonic cell transcription factor 1; PIWIL4, PIWI-like protein 4; ID4, inhibitor of DNA binding 4; FGFR3, fibroblast growth factor receptor 3; KIT, tyrosine kinase receptor.

a Analysis not controlled for age.

b Reduced spermatogonial pool below the reference range (S/T *Z*-score<3SD), 0 = no, 1 = yes.

c Depleted spermatogonia pool (S/T *Z*-score<−7SD), 0 = no, 1 = yes. *P*-values shown in bold are statistically significant.

### Effect of chemotherapy exposure and spermatogonia pool depletion on early SSC

To assess the effects of chemotherapy exposure on spermatogonia at distinct differentiation stages, the impact of cumulative doses of alkylating agents (CED) and anthracyclines (DIE) on spermatogonial marker protein expression was analysed (Table 1, Supplementary Fig. S4). Patients were exposed to a median dose of CED at 4.2 g/m^2^ (range: 0.5–16.0 g/m^2^) and DIE at 200 mg/m^2^ (range: 40–500 mg/m^2^). Increased exposure to CED decreased the number of all spermatogonial subpopulations in a dose-dependent manner, with a significant decrease in two marker expressions, namely the spermatogonia expressing ID4 and KIT proteins controlled for age (Table 1, Supplementary Fig. S4). Additionally, increased DIE exposure correlated significantly with decreased KIT protein controlled for age (Table 1). Similarly, increased exposure to CED and DIE significantly correlated with decreased S/T *Z*-score values (rho −0.778, *P* < 0.001, and −0.454, *P* = 0.002, respectively) and increased exposure to DIE with older age (rho 0.367, *P* = 0.013) (data not shown).

We then explored whether the size of the spermatogonia pool affects numbers of spermatogonia at distinct early stages. The S/T *Z*-score was associated with the number of spermatogonia expressing ID4 and KIT (Supplementary Fig. S5). When patients were grouped into those with and without a depleted spermatogonia pool, reduced numbers of spermatogonia expressing ID4 and FGFR3 were observed in testicular samples with a depleted spermatogonial pool (S/T *Z*-score <−7SD) compared to those without depletion (Table 2). Further, a reduced spermatogonial pool (S/T *Z*-score <−3SD) correlated significantly with fewer spermatogonia expressing ID4 and KIT proteins. No correlation was observed with protein expression of UTF1 or PIWIL4 representing the earliest/naïve state of spermatogonia. No difference in median age was observed between the two groups, but patients with a depleted pool had been exposed to higher median doses of CED and DIE (Table 2).

**Table 2.** Clinical characteristics, spermatogonia quantity, and spermatogonia marker expression in the 10 prepubertal testicular samples with depleted (S/T *Z*-score<−7 SD) and 35 with non-depleted (S/T *Z*-score ≥−7 SD) spermatogonia pool.

	Spermatogonia pool				
Characteristics	Non-depleted N = 35		Depleted N = 10		***P*-value** ^a^
	n	Median (range)	n	Median (range)	
**Clinical characteristics**					
Age (yrs)	35	6.1 (0.6–13.1)	10	6.6 (1.3–10.0)	0.904
CED (g/m^2^)	35	0 (0–7.0)	10	4.6 (2.0–16.0)	**<0.001**
DIE (mg/m^2^)	35	0 (0–456)	10	200 (40–500)	**<0.001**
**Spermatogonia quantity**					
S/T	35	0.93 (0.05–16.4)	10	0.04 (0.02–0.06)	**<0.001**
S/T *Z*-score (SD)	35	−2.1 (−6.9 to −1.9)	10	−13.6 (−23.4 to −7.2)	**<0.001**
**Spermatogonia marker expression (cell/area mm^2^)**					
UTF1	31	219 (52–749)	10	247 (0–715)	0.569
PIWIL4	35	121 (0–287)	10	45(0–221)	0.154
ID4	34	181 (0–695)	10	48(0–550)	**0.035**
FGFR3	33	134 (0–394)	10	56 (0–658)	**0.048**
KIT	35	0 (0–245)	10	0 (0–0)	0.139

CED, cumulative cyclophosphamide equivalent dose; DIE, doxorubicin isoequivalent dose equivalent; S/T, spermatogonial numbers per round tubular cross-section; UTF1, undifferentiated embryonic cell transcription factor 1; PIWIL4, PIWI-like protein 4; ID4, inhibitor of DNA binding 4; FGFR3, fibroblast growth factor receptor 3; KIT, tyrosine kinase receptor.

a *P*-values were derived from Mann–Whitney *U*-tests. *P*-values shown in bold are statistically significant.

## Discussion

Despite existing classification models, the knowledge regarding human spermatogonia, including SSC development and maturation in child testes is still limited. Full understanding will require the integration of multiple data types including shared data repositories, biobank samples, and fertility preservation material. Here, we aimed to combine previously obtained scRNA-seq data of spermatogonia within the normal child testis (Guo *et al.*, 2020), complemented by immunofluorescence staining of early spermatogonial markers in prepubertal testicular material from biobank and patients undergoing cancer treatment. Spermatogonial subpopulations were analysed both at baseline and after chemotherapy-induced reduction of the spermatogonial pool. This study is the first to confirm distinct transcriptional and developmental spermatogonial states at the protein level in clinical fertility preservation samples. Chemotherapy-induced spermatogonial reduction was shown to alter developmental spermatogonial states and was associated with a reduced number of spermatogonia expressing markers of later states. Undifferentiated spermatogonial subtypes expressing the protein markers UTF1 and PIWIL4 did not correlate with spermatogonial pool reduction, suggesting they may represent a quiescent ‘reserve’ stem cell pool that is more resistant to chemotherapy-induced reduction.

Furthermore, we confirm previously reported germline development in child testis using scRNA-seq data (Guo *et al.*, 2020). The distribution of most undifferentiated spermatogonia types (state 0–1) for all six donors aged 0–17 years showed substantial overlap, demonstrating overall consistency between donors. However, remarkable differences were observed in more advanced spermatogonia states which were absent in boys younger than 7 years. These findings suggest that the transition to more differentiated spermatogonia states is closely linked to age, highlighting a critical developmental window. Specific marker expressions associated with these advanced states provide further insight into the maturation process of spermatogonia. For example, genes such as *FGFR3*, and *KIT* were up-regulated while *ID4* was down-regulated in advanced ages, indicating their potential role in the progression of spermatogenesis. This raises important questions about the regulatory mechanisms governing spermatogonial differentiation and the implications for fertility in younger individuals.

Low doses of chemo- or radiotherapy may deplete the pool of differentiating spermatogonia, while reserve SSCs survive, and after initiation of pubertal development spermatocytes and spermatids continue their maturation into sperm (Meistrich, 2013). The potential for recovery of sperm production after a cytotoxic insult depends on the ability of mitotically quiescent stem spermatogonia to survive and resume mitotic activity and to produce differentiating spermatogonia. If the damage is severe, for example, because of a high cumulative dose of alkylating agent or irradiation, all the reserve SSCs may commit to apoptosis and the patient will become permanently infertile.

Experimental studies under controlled conditions have revealed a negative effect of radiation and high-dose chemotherapy on spermatogenesis in non-human primates. In 2002, de Rooij *et al.* (2002) assessed the long-term reproductive outcomes in male rhesus macaques, showing that total-body irradiation of 4–8.5 Gy during prepuberty (2–4 years of age) resulted in incomplete spermatogenic recovery when evaluated 3–8 years later. While higher radiation doses (>8 Gy) were associated with total germ cell depletion, lower doses resulted in spermatogonial repopulation (de Rooij *et al.*, 2002). In an *in vitro* context, Jahnukainen *et al.* (2007) observed similar effects when testicular tissue from immature monkeys was exposed to 0–4 Gy *in vitro* and subsequently xenotransplanted into nude mice for 4 months. The grafted testicular tissue exhibited a dose-dependent depletion of spermatogonia after 4 months. Utilizing a comparable approach, the impact of busulfan (alkylating agent) treatment on host mice bearing xenotransplanted non-human primate testicular fragments was evaluated (Jahnukainen *et al*., 2006). Histological analysis 1-month post-transplantation revealed a marked depletion of germ cells; however, undifferentiated spermatogonia (*A*_dark_) persisted despite treatment (Jahnukainen *et al.* 2006). Complementary research conducted in adult rhesus macaques revealed similar results, showing the negative effects of high-dose busulfan treatment on non-human primate testes (Hermann *et al.*, 2007).

Similarly, in human testes, spermatogonia have been shown to be susceptible to such depletion at all stages of life (Skinner *et al.*, 2017). In the present study we monitored the spermatogonia subsets in testicular samples following chemotherapy-induced reduction of the spermatogonial pool. There was a significant decrease in all spermatogonial subpopulations, but the extent of the decrease varied between spermatogonial states. Reduced expression of ID4 (states 0–1), FGFR3 (states 0–2), and KIT (state 4) proteins correlated with pool reduction. This may be attributed to their overall roles in regulating various cellular processes, such as cell proliferation (FGFR3, KIT), cell differentiation (FGFR3, ID4), and apoptosis/survival (FGFR3, KIT, and ID4), which makes them particularly sensitive to gonadotoxic exposure.

In contrast, spermatogonia expressing markers of the most naïve, undifferentiated spermatogonia, such as UTF1 and PIWIL4, were more resistant, and their decrease did not correlate with reduction of the spermatogonial pool. Spermatogonia expressing UTF1 and PIWIL4 proteins may potentially represent a quiescent reserve stem cell pool. Although cell cycle activity was not tested in this study, our results suggest that PIWIL4- and UTF1-expressing cells are more resistant to highly gonadotoxic treatments. These findings support previous observations indicating that PIWIL4-expressing spermatogonia identified in infants and UTF1-positive spermatogonia found in juvenile testes constitute a reserve population of undifferentiated, quiescent, or slow-cycling spermatogonia (Guo *et al.*, 2020). Conformingly, the UTF1-positive cells have shown to persist from infancy through adulthood (Guo *et al.*, 2020). Current strategies to preserve the reproductive potential of prepubertal patients focus on the cryopreservation of testicular tissue samples containing reserve SSCs. Therefore, understanding both the quantity and functionality of spermatogonial cells in these tissue samples is crucial for developing personalized fertility restoration strategies in the future. The present observation that spermatogonia from five distinct developmental states may vary in their sensitivity to gonadotoxic insult suggests that only a subset of spermatogonia in testicular tissue are potentially quiescent ‘reserve’ stem cells.

Some limitations need to be recognized when interpreting the present findings. The study population was heterogeneous in terms of age and treatment exposure. Moreover, the impact of specific cancer treatments could not be individually assessed, and limited tissue availability reduced the statistical power of the study. Due to limited tissue availability, repeated double or triple immunofluorescence staining could not be performed. As a result, the correlations between the expression of different spermatogonial markers can only be considered indicative trends. Testicular tissue samples were obtained from the internal biobank of Karolinska University Hospital and were considered normal for inclusion if no testicular pathology was reported. However, detailed information on prior medical treatments or testicular volumes for the patients in this biobank was unavailable. Finally, our study includes several statistical analyses, but the results are not adjusted for multiple comparisons.

In conclusion, distinct protein expression patterns of early spermatogonia states were observed in samples with chemotherapy-induced reduction of the spermatogonial pool. Reduced expression of ID4 (states 0–1), FGFR3 (states 0–2), and KIT (state 4) proteins correlated with a reduced spermatogonial pool, whereas markers of the most naïve undifferentiated spermatogonia, such as UTF1 and PIWIL4, were more resistant to cytotoxic insult and pool reduction. These subtypes may potentially represent a quiescent ‘reserve’ stem cell pool required for spermatogenic recovery. Identifying such reserve stem cells could provide a valuable method for assessing the fertility potential of testicular tissue collected for preservation.

## Supplementary Material

deaf103_Supplementary_Figure_S1

deaf103_Supplementary_Figure_S2

deaf103_Supplementary_Figure_S3

deaf103_Supplementary_Figure_S4

deaf103_Supplementary_Figure_S5

deaf103_Supplementary_Table_S1

deaf103_Supplementary_Table_S2

## Data Availability

The raw data related to patient samples included in this study are available upon request from the corresponding author. The raw gene expression matrices were retrieved from the Gene Expression Omnibus (GEO) under accession numbers GSE134144 and GSE120508.
