# Supplementary material for: Early transcriptional states of spermatogonia and marker expressions in the prepubertal human testis following chemotherapy-induced depletion
Source: Hum Reprod. 2025 Jun 7;40(8):1467–75. doi: 10.1093/humrep/deaf103 (PMC12314143; doi:10.1093/humrep/deaf103)
Supplement: deaf103_Supplementary_Figure_S1 [file deaf103_supplementary_figure_s1.pdf]

Human Reproduction, 2025, 1–1
https://doi.org/10.1093/humrep/deaf103
Supplementary Data

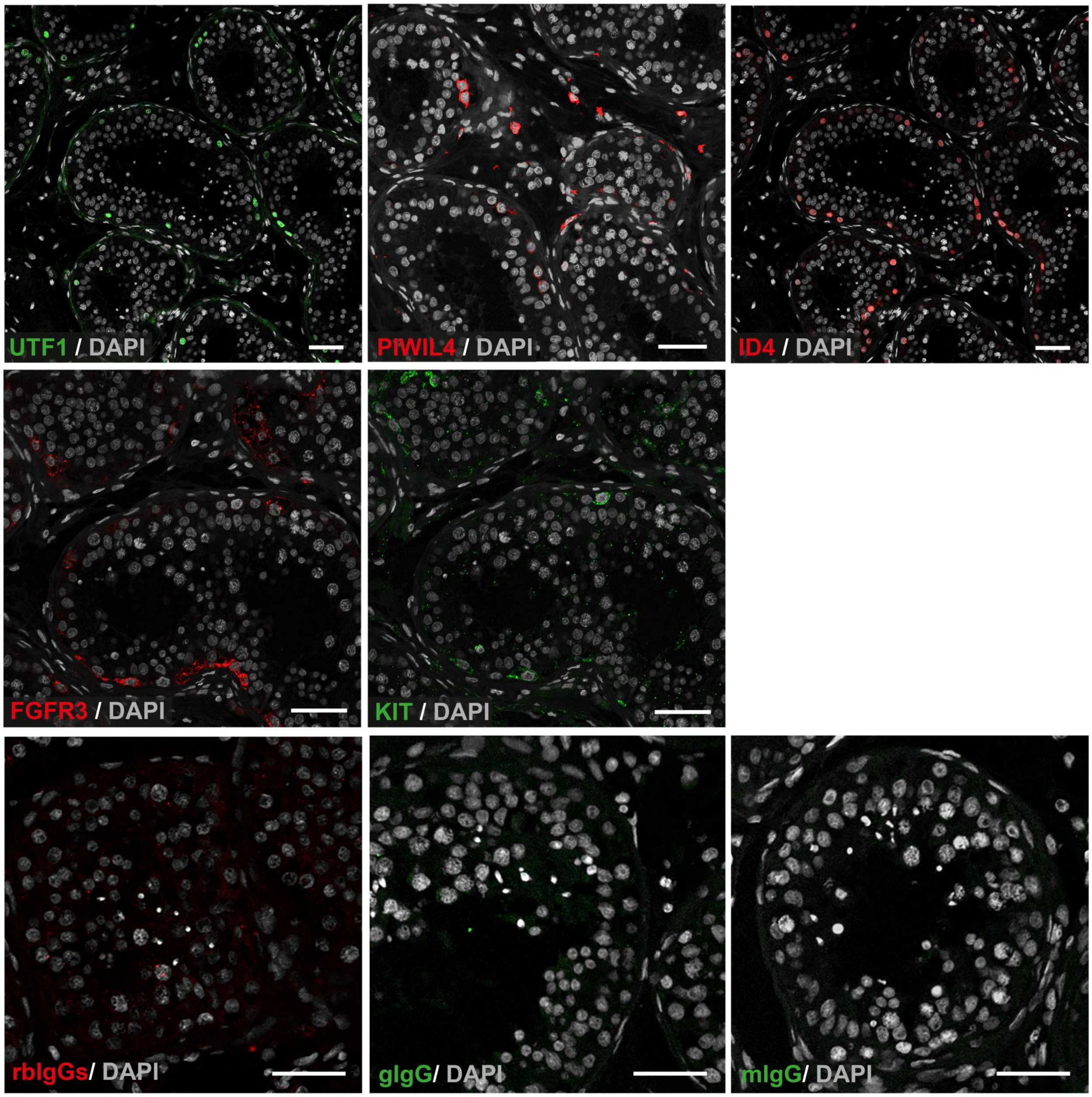

Supplementary Figure S1. Representative immunofluorescence staining images for spermatogonia marker UTF1 (green), PIWIL4 (red), ID4 (red), FGFR3 (red), and KIT (green) in adult testes (serving as positive controls). Cell nuclei are stained with DAPI (grey). Rabbit IgGs (rbIgGs), goat IgGs (gIgGs), and mouse IgGs (mIgGs) were used as negative controls. Scale bars: 50 μm, inserts: 10 μm. UTF1, undifferentiated embryonic cell transcription factor 1; PIWIL4, PIWI-like protein 4; ID4, inhibitor of DNA binding 4; FGFR3, fibroblast growth factor receptor 3; KIT, tyrosine kinase receptor.
