# Supplementary material for: Early transcriptional states of spermatogonia and marker expressions in the prepubertal human testis following chemotherapy-induced depletion
Source: Hum Reprod. 2025 Jun 7;40(8):1467–75. doi: 10.1093/humrep/deaf103 (PMC12314143; doi:10.1093/humrep/deaf103)
Supplement: deaf103_Supplementary_Figure_S2 [file deaf103_supplementary_figure_s2.pdf]

OXFORD
human reproduction

Human Reproduction, 2025, 1–1
https://doi.org/10.1093/humrep/deaf103
Supplementary Data

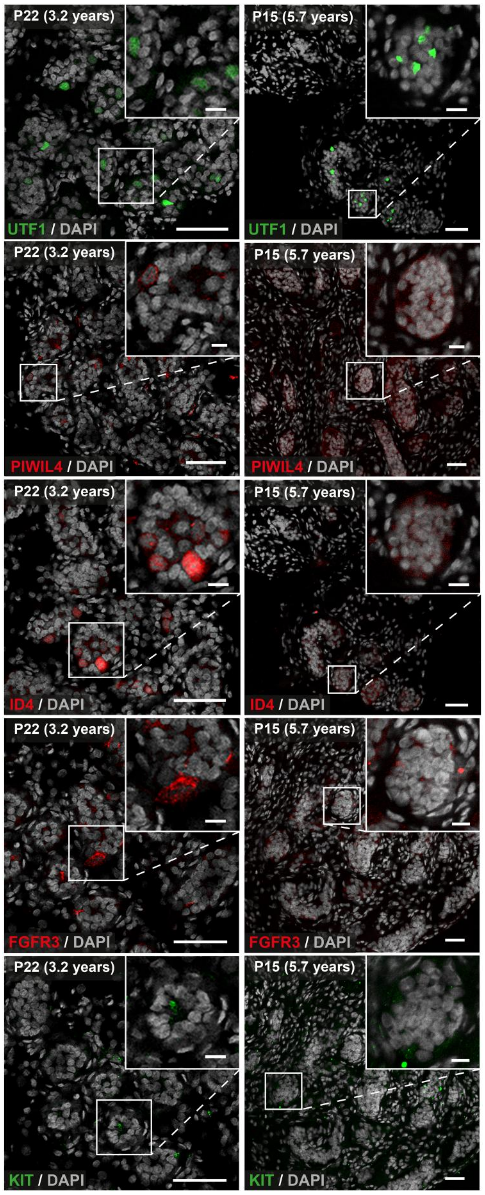

Supplementary Figure S2. Representative immunofluorescence staining images of spermatogonial markers UTF1 (green), PIWIL4 (red), ID4 (red), FGFR3 (red), and KIT (green) are shown for testes samples from a 3.2-year-old patient with a non-depleted spermatogonial pool (S/T *Z*-score: −3.06) and a 5.7-year-old patient with a depleted spermatogonial pool (S/T *Z*-score: −8.68) (P22 and P15, respectively). Cell nuclei are counterstained with DAPI (grey). Scale bars: 50 μm, inserts: 10 μm. UTF1, undifferentiated embryonic cell transcription factor 1; PIWIL4, PIWI-like protein 4; ID4, inhibitor of DNA binding 4; FGFR3, fibroblast growth factor receptor 3; KIT, tyrosine kinase receptor.
