# Supplementary material for: Early transcriptional states of spermatogonia and marker expressions in the prepubertal human testis following chemotherapy-induced depletion
Source: Hum Reprod. 2025 Jun 7;40(8):1467–75. doi: 10.1093/humrep/deaf103 (PMC12314143; doi:10.1093/humrep/deaf103)
Supplement: deaf103_Supplementary_Figure_S3 [file deaf103_supplementary_figure_s3.pdf]

OXFORD
human reproduction
Human Reproduction, 2025, 1–1
https://doi.org/10.1093/humrep/deaf103
Supplementary Data

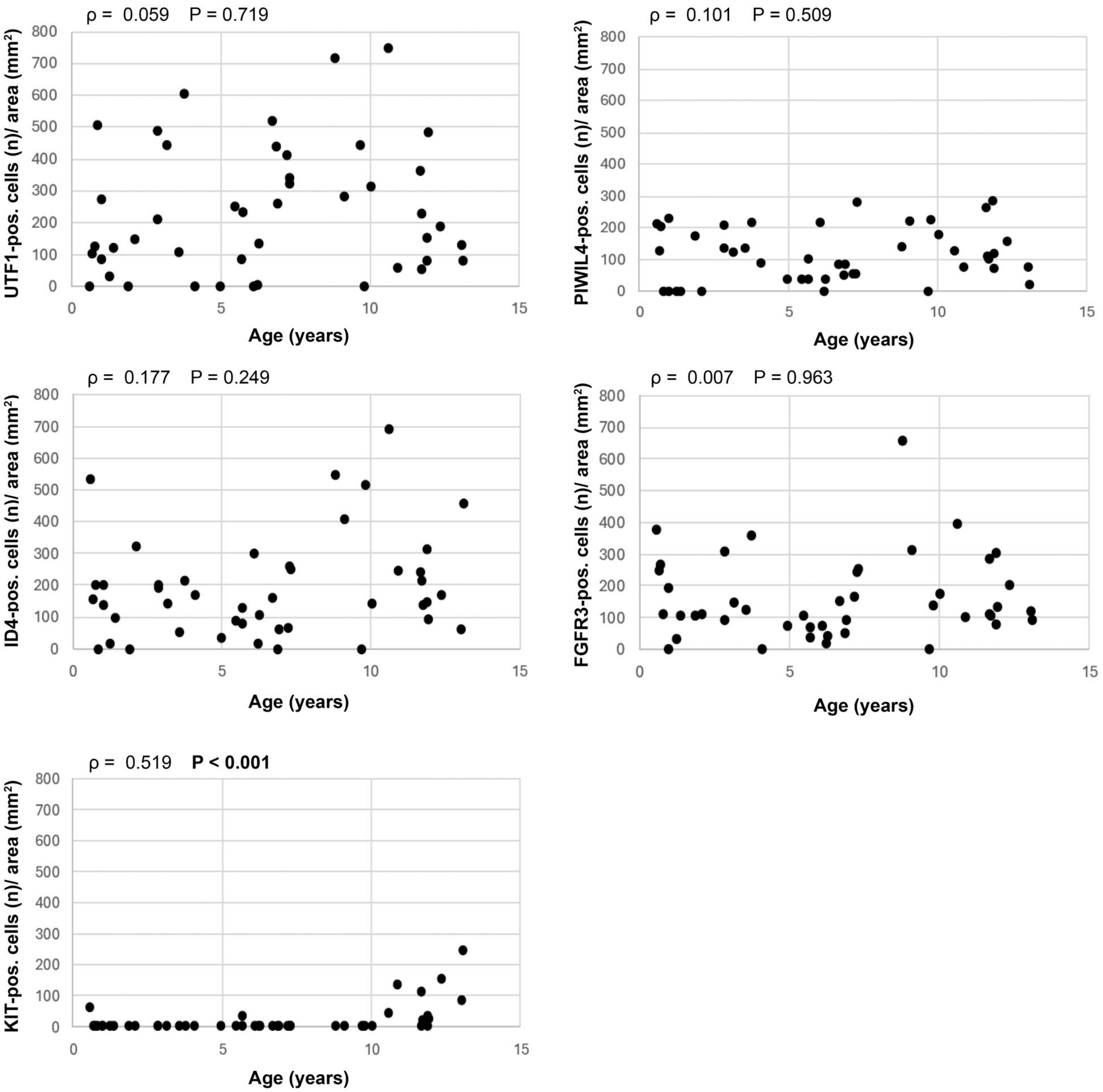

Supplementary Figure S3. Correlation analysis of spermatogonial marker expression, including UTF1, PIWIL4, ID4, FGFR3, and KIT, was performed by quantifying positive cells per area and plotting these values against age in years. Spearman correlation coefficients (ρ) and the *P*-values are presented. UTF1, undifferentiated embryonic cell transcription factor 1; PIWIL4, PIWI-like protein 4; ID4, inhibitor of DNA binding 4; FGFR3, fibroblast growth factor receptor 3; KIT, tyrosine kinase receptor.
