# Supplementary material for: Early transcriptional states of spermatogonia and marker expressions in the prepubertal human testis following chemotherapy-induced depletion
Source: Hum Reprod. 2025 Jun 7;40(8):1467–75. doi: 10.1093/humrep/deaf103 (PMC12314143; doi:10.1093/humrep/deaf103)
Supplement: deaf103_Supplementary_Table_S1 [file deaf103_supplementary_table_s1.pdf]

OXFORD
human reproduction
Human Reproduction, 2025, 1–1
https://doi.org/10.1093/humrep/deaf103
Supplementary Data

Supplementary Table S1. Clinical information for the 45 patients included in the study.

ID	Age years	Indication for biopsy	Diagnosis	Cancer Diagnosis	Exposure to chemotherapy	CED mg/m^2	DIE mg/m^2	S/T Z-score
P1	0.7	Fertility preservation	Leukaemia	Yes	No	0	0	−3.62
P2	0.7	Fertility preservation	Leukaemia	Yes	No	0	0	−1.60
P3	0.8	Fertility preservation	Leukaemia	Yes	No	0	0	−3.82
P4	2.9	Fertility preservation	Leukaemia	Yes	No	0	0	−6.52
P5	2.9	Fertility preservation	Solid tumour	Yes	No	0	0	−6.00
P6	13.1	Fertility preservation	Solid tumour	Yes	No	0	0	−0.82
P7	10.6	Fertility preservation	Leukaemia	Yes	Yes	0	80	−0.31
P8	11.9	Fertility preservation	Leukaemia	Yes	Yes	0	80	−1.23
P9	3.8	Fertility preservation	Leukaemia	Yes	Yes	0	336	−0.45
P10	7.3	Fertility preservation	Leukaemia	Yes	Yes	0	388	−2.55
P11	11.7	Fertility preservation	Leukaemia	Yes	Yes	0	388	−1.36
P12	11.9	Fertility preservation	Leukaemia	Yes	Yes	0	456	−1.21
P13	6.7	Fertility preservation	Leukaemia	Yes	Yes	488	101	−4.64
P14	7.3	Fertility preservation	Leukaemia	Yes	Yes	1000	80	−3.09
P15	5.7	Fertility preservation	Leukaemia	Yes	Yes	2000	60	−8.68
P16	8.8	Fertility preservation	Leukaemia	Yes	Yes	2000	230	−23.4
P17	9.7	Fertility preservation	Leukaemia	Yes	Yes	2000	232	−2.29
P18	7.2	Fertility preservation	Leukaemia	Yes	Yes	2000	260	−4.64
P19	9.1	Fertility preservation	Leukaemia	Yes	Yes	2200	40	−8.21
P20	11.7	Fertility preservation	Leukaemia	Yes	Yes	2200	120	−3.20
P21	3.5	Fertility preservation	Solid tumour	Yes	Yes	4200	0	−4.64
P22	3.2	Fertility preservation	Solid tumour	Yes	Yes	4200	0	−3.06
P23	5.5	Fertility preservation	Solid tumour	Yes	Yes	4200	135	−4.39
P24	6.9	Fertility preservation	Leukaemia	Yes	Yes	4400	160	−18.81
P25	6.9	Fertility preservation	Leukaemia	Yes	Yes	4400	120	−18.29
P26	6.3	Fertility preservation	Solid tumour	Yes	Yes	4814	200	−17.6
P27	10.0	Fertility preservation	Leukaemia	Yes	Yes	6976	280	−7.19
P28	12.4	Fertility preservation	Leukaemia	Yes	Yes	6976	298	−6.85
P29	5.0	Fertility preservation	Leukaemia	Yes	Yes	7600	200	−8.68
P30	1.3	Fertility preservation	Solid tumour	Yes	Yes	10 500	250	−9.54
P31	6.2	Fertility preservation	Solid tumour	Yes	Yes	16 000	500	−20.48
P32	0.6	Biobank sample	–	No	No	0	0	−1.24
P33	1	Biobank sample	–	No	No	0	0	−1.92
P34	1	Biobank sample	–	No	No	0	0	−1.11
P35	1.4	Biobank sample	–	No	No	0	0	−2.31
P36	1.9	Biobank sample	–	No	No	0	0	−1.04
P37	2.1	Biobank sample	–	No	No	0	0	1.52
P38	4.1	Biobank sample	–	No	No	0	0	0.22
P39	5.7	Biobank sample	–	No	No	0	0	−2.44
P40	6.1	Biobank sample	–	No	No	0	0	−2.49
P41	9.8	Biobank sample	–	No	No	0	0	1.65
P42	10.9	Biobank sample	–	No	No	0	0	0.45
P43	11.7	Biobank sample	–	No	No	0	0	0.75
P44	11.9	Biobank sample	–	No	No	0	0	−2.11
P45	13.1	Biobank sample	–	No	No	0	0	1.91

Samples P1–P31 were collected through the NORDFERTIL fertility preservation program, while samples P32–P45, with no reported testicular pathology, were obtained from the pathology biobank.
CED, cumulative cyclophosphamide equivalent dose; DIE, doxorubicin isoequivalent dose equivalent; S/T spermatogonia numbers per round tubular cross-section.
