# Supplementary material for: Early transcriptional states of spermatogonia and marker expressions in the prepubertal human testis following chemotherapy-induced depletion
Source: Hum Reprod. 2025 Jun 7;40(8):1467–75. doi: 10.1093/humrep/deaf103 (PMC12314143; doi:10.1093/humrep/deaf103)
Supplement: deaf103_Supplementary_Table_S2 [file deaf103_supplementary_table_s2.pdf]

Human Reproduction, 2025, 1–1
https://doi.org/10.1093/humrep/deaf103
Supplementary Data

Supplementary Table S2. The key features, sources, and applications of primary and secondary antibodies used in immunofluorescence staining, as documented by the Human Protein Atlas (https://www.proteinatlas.org/).

Protein	Host Species	Antibody concentration	Antibody dilution	Catalogue number & source	Main localization
DDX4	mouse	1 mg/ml	1:200	ab27591, Abcam, Cambridge, UK	Germ cells (Cytoplasm)
KIT	goat	5–15 μg/ml	1:25	AF332, R&D systems, Minneapolis, MN, USA	Spermatogonia & Spermatocytes (Cytoplasm)
FGFR3	rabbit	1 mg/ml	1:50	C51F2, Cell Signalling Technology, Leiden, The Netherlands	Spermatogonia (Cytoplasm)
ID4	rabbit	0.05 mg/ml	1:100	M107, Cal Bioreagents, San Mateo, CA, USA	Spermatogonia Intracellular (Nucleoplasm)
PIWIL4	rabbit	0.4 mg/ml	1:100	PA036588, Sigma–Aldrich, St. Louis, MO, USA	Spermatogonia Intracellular (Nucleoplasm Mitochondria)
UTF1	mouse	1 mg/ml	1:50	MAB4337, Millipore, Merck Life Science AB, Solna, Sweden	Spermatogonia (Intracellular)
Mouse IgG	mouse	0.4 mg/ml	1:100	MAB0031, R & D systems	N/A
Rabbit IgG	rabbit	1.775 mg/ml	1:200	ab172730, Abcam	N/A
Goat IgG	goat	1 mg/ml	1:200	AB-108-C, R&D Systems	N/A
Cy3	rabbit	1.5 mg/ml	1:500	711-166-152, Jackson Immuno Research, West Grove, PA, USA	N/A
Alexa Fluor 488	mouse	1.5 mg/ml	1:500	715-546-150, Jackson Immuno Research	N/A
Alexa Fluor 568	rabbit	2 mg/ml	1:500	A-10042, Invitrogen, Waltham, MA, USA	N/A
Alexa Fluor 488	mouse	2 mg/ml	1:500	A-21202, Invitrogen	N/A
Alexa Fluor 488	goat	2 mg/ml	1:500	A-11055, Invitrogen	N/A

DDX4, DEAD-box helicase 4; KIT, tyrosine kinase receptor; FGFR3, fibroblast growth factor receptor 3; ID4, inhibitor of DNA binding 4; UTF1, undifferentiated embryonic cell transcription factor 1; PIWIL4, PIWI-like protein 4.

© The Author(s) 2025. Published by Oxford University Press on behalf of European Society of Human Reproduction and Embryology.
This is an Open Access article distributed under the terms of the Creative Commons Attribution License (https://creativecommons.org/licenses/by/4.0/), which permits unrestricted reuse, distribution, and reproduction in any medium, provided the original work is properly cited.
Human Reproduction, 2025, 00, 1–16
https://doi.org/10.1093/humrep/deaf103
Original Article
